# MabCent: Arctic marine bioprospecting in Norway

**DOI:** 10.1007/s11101-012-9239-3

**Published:** 2012-05-30

**Authors:** Johan Svenson

**Affiliations:** SmallStruct, Department of Chemistry, University of Tromsø, Breivika, 9037 Tromsø, Norway

**Keywords:** Pharmacognosy, Arctic, Cold adaption, Psychrophile, Screening, Bioprospecting, Norway, MabCent

## Abstract

The deep waters surrounding the coastline of the northern parts of Norway represent an exciting biotope for marine exploration. Dark and cold Arctic water generates a hostile environment where the ability to adapt is crucial to survival. These waters are nonetheless bountiful and a diverse plethora of marine organisms thrive in these extreme conditions, many with the help of specialised chemical compounds. In comparison to warmer, perhaps more inviting shallower tropical waters, the Arctic region has not been as thoroughly investigated. MabCent is a Norwegian initiative based in Tromsø that aims to change this. Since 2007, scientists within MabCent have focussed their efforts on the study of marine organisms inhabiting the Arctic waters with the long term goal of novel drug discovery and development. The activities of MabCent are diverse and range from sampling the Arctic ice shelf to the chemical synthesis of promising secondary metabolites discovered during the screening process. The current review will present the MabCent pipeline from isolation to identification of new bioactive marine compounds via an extensive screening process. An overview of the main activities will be given with particular focus on isolation strategies, bioactivity screening and structure determination. Pitfalls, hard earned lessons and the results so far are also discussed.

## Introduction

With one of the longest coastlines in the world it is not surprising that Norway is a country with a rich history in both marine exploration and exploitation. The discovery of massive oil and gas deposits in the late 1960s had a huge impact, rapidly transforming a humble nation dependent on fishing and shipping to a very wealthy country. The initial discoveries were made in the North Sea and have been followed by significant finds in the Barents Sea making Norway a developed country with a high standard of living. Being well aware of the fact that a national economy based on the petroleum industry is not long term viable the government is now attempting to convert some of its revenue into sustainable industries capable of excelling in the post-petroleum era. As a result, several initiatives to generate internationally competitive Centres for Research based Innovation (CRI) have been launched by the Norwegian research council since 2007. Furthermore, in 2009, the Norwegian government launched a national strategy entitled “Marine bioprospecting—a source of new and viable wealth creation”, providing funding for a national infrastructure and research within marine natural products and drug discovery.

**Fig. 1:**
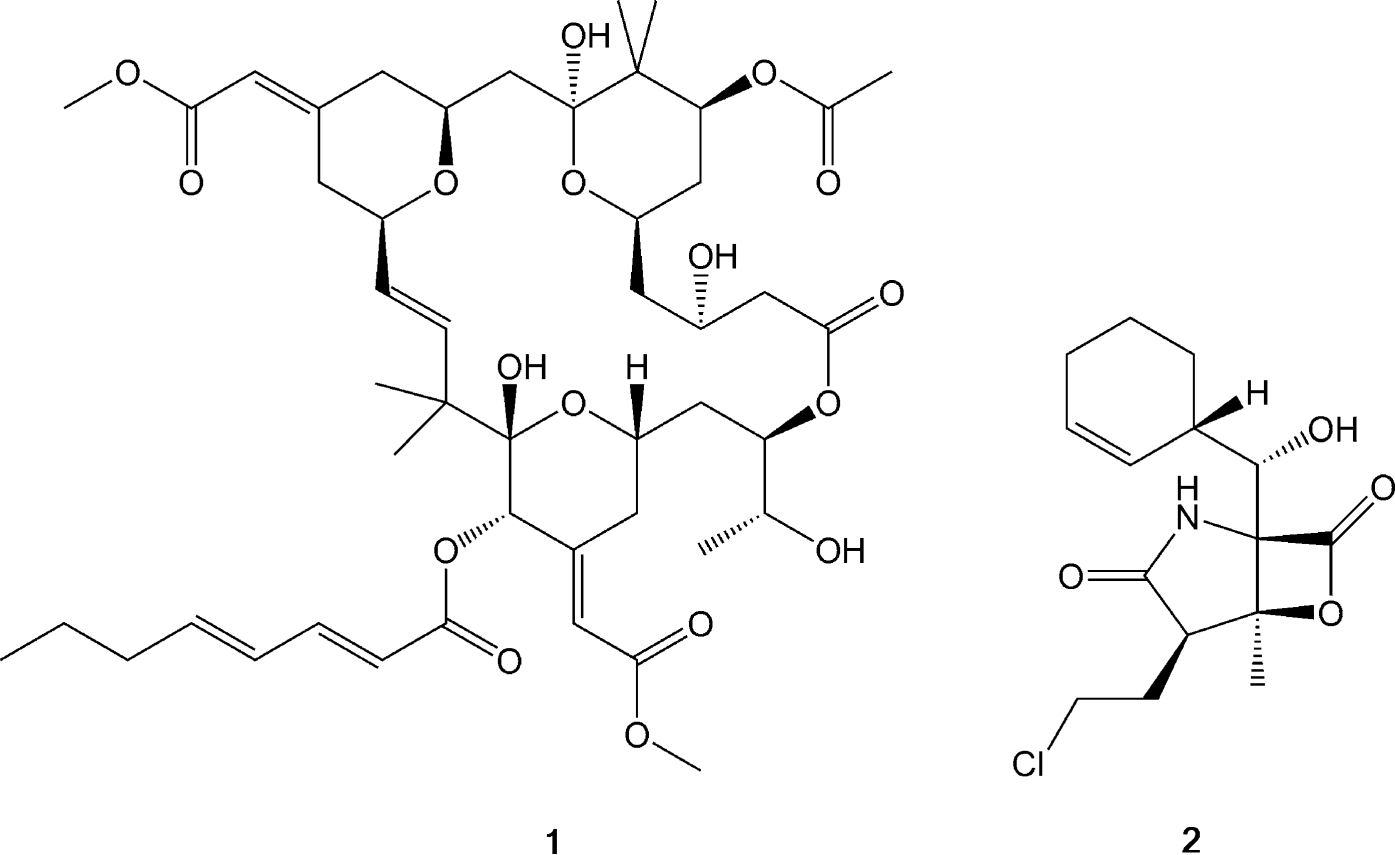
Examples of highly potent marine compounds currently undergoing clinical trials. The challenging macrocyclic lactone bryostatin-1 (**1**) with its 11 stereocentres was only recently prepared synthetically (Keck et al. 2011). The unusual β-lactone salinosporamide A isolated from the halophilic actinomycete *Salinispora* (**2**) is a potent orally active proteasome inhibitor (Chauhan et al. 2005)

Norwegian scientists are for this reason once more turning to the ocean for inspiration. The deep fjords and the open oceans surrounding the Norwegian mainland are cold and dark and organisms willing to survive here must adapt to the extreme conditions. They do so by producing a range of secondary metabolites and specialised proteins providing them with advantages and protection against predation and microbial intruders (Kubanek et al. 2003; Matz et al. 2008). Such compounds have been chemically optimised during millions of years of evolution and represent entities with significant potential for several industrial and medical applications (Molinski et al. 2009; Mayer et al. 2010). Nearly two thirds of the commercial pharmaceuticals originate from natural products (Cragg et al. 2009). There is a precedence for marine metabolites as approved therapeutics. Ziconotide (Prialt^®^) is a synthetic analogue of a naturally occurring 25-amino acid peptide, ω-Conotoxin MVIIA, which is isolated from the venom of the piscivorous cone snail *Conus magus* (Olivera et al. 1985; Terlau and Olivera 2004)*.* It was the first marine-derived compound approved by FDA in December 2004 as a potent analgesic (Garber 2005). Several other promising leads such as bryostatin-1 (compound **1** in Fig. 1) from *Bugula neritina* (Pettit et al. 1982) with subnanomolar binding to protein kinase C as well as numerous other biological activities (Mutter and Wills 2000) and salinosporamide A, active against multiple myeloma (Chauhan et al. 2008; Fenical et al. 2009), shown as compound **2** in Fig. 1 are currently under development.

As of 2011, six marine derived drugs have been approved by FDA and one by the European Union. Thirteen others are in various stages of clinical trials (Mayer et al. 2010, 2011).

**Fig. 2:**
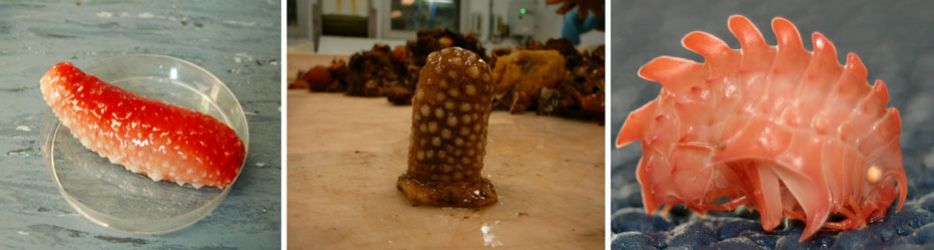
The deep cold waters are rich in biodiversity and shown are three examples of diverse and colourful inhabitants. From the left *Parastichopus tremulus*, *Hormathia nodosa* and the amphipode *Epimeria loricata* to the right.^ ©^Johan Svenson & Robert Johansen, UiT/Marbank

Despite its abundance of marine organisms, Norway has done relatively little towards studying their contents. MabCent is a Norwegian initiative headed by Professor Trond Ø. Jørgensen based in Tromsø that aims to change this. The centre for research based innovation has been focussed on studying Arctic marine organisms as these represent a group of organisms that have not been well studied and have the potential to display interesting biochemistries due their adaption to living conditions near, and below the freezing point of water (Fig. 2).

Easy access to organisms is a significant advantage and it is not coincidental that the MabCent endeavour is based at the University of Tromsø (UiT), the northernmost university in the world at 70° north, some 300 km north of the Arctic Circle. UiT was particularly well suited for this task with all the essential competence and infrastructure (ranging from Arctic research vessels and marine biologist to structural chemists) readily available. With a gross budget of nearly 23 million Euros from the Norwegian research council over a period of 8 years and additional financial support from UiT and commercial partners the project management have sufficient funding and time to serve its main purpose: “to find and develop high-value bioactive products for scientific and commercial exploitation by screening compounds from Arctic organisms”. All the activities are based in Tromsø but there is also collaboration with the newly established Marine Biodiscovery Centre in Aberdeen, Scotland.

Nearly sixty staff members are associated with the MabCent activities and the centre has its own Ph.D. programme and an external scientific advisory board. In addition, four commercial partners are also participating in the research (Table 1). These partners contribute both financially and scientifically by providing key competences within their specialised field of expertise. For an annual fee the companies have the right to develop promising leads into commercial products according to a first right of refusal agreement. Four platforms manage the main activities and their responsibilities are listed in Table 1.

**Table 1:** Commercial partners and central platforms within the MabCent organisation

Commercial partner	Field
Lytix Biopharma	Anticancer
Biotec Pharmacon	Antibacterial/Enzymes/Immunomodulation
Pronova BioPharma	Type-II Diabetes/Anti-inflammation
ABC Bioscience	Antioxidants

Platform	Responsibility
Marbank	Organism collection/Biobank
Marbio	Screening and Purification
NorStruct	Structure elucidation macromolecules
SmallStruct	Structure elucidation small molecules

## Collection

**Fig. 3:**
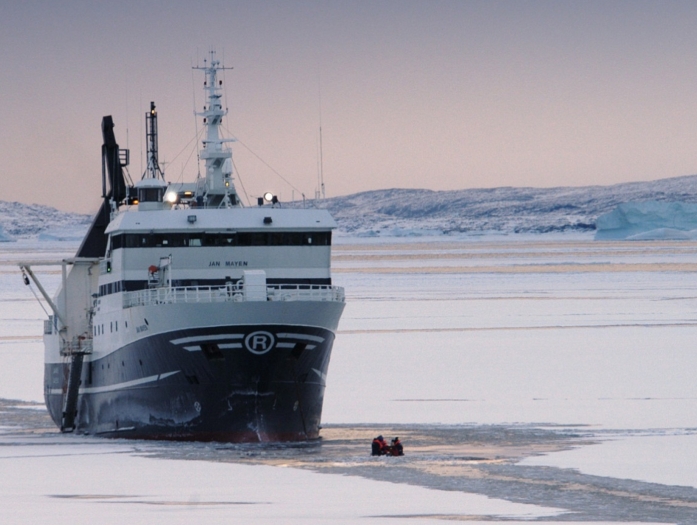
Access to sturdy and modern research vessels with ice breaking capacity enables sampling in the extreme environments studied. Shown above is the 60 metre Helmer Hanssen (previously known as Jan Mayen) on a collection cruise in hostile surroundings. © TUNU-MAFIG, UiT

The availability of biological material is paramount, and the cooperation with the marine biobank Marbank is therefore essential. Marbank has the national responsibility for the collection and preservation of marine resources/organisms for scientific research, commercial opportunities and exploitation purposes. Three research vessels are at Marbank’s disposal and they are purposefully designed for the task. The largest one, *Helmer Hanssen*, at 60 m is a modern science platform capable of long journeys into the pack ice and capable of collecting samples in several thousand metres of water (Fig. 3). While most of the ocean waters on the globe can be regarded as dark and cold and therefore potentially rich in psychrophilic organisms, MabCent has mainly focused on Arctic waters with temperatures near or below zero degrees. The sampling stretches from the pack ice north of Svalbard, to the northern Norwegian coast line. Sub-zero degree water temperatures can also be found in deep water pockets in several of the fjords along the northern coastline. The material is collected during many annual excursions.

**Fig. 4:**
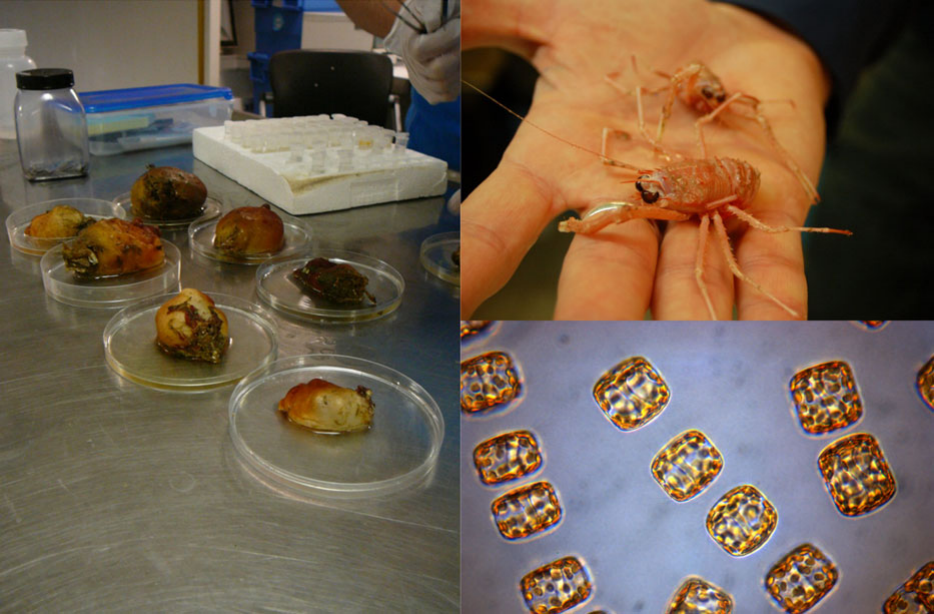
DNA is collected from each collected species, both microorganisms and benthic. Shown to the *right* are *Munida sarsi* lobsters and *Porosia glacialis* microalgae.^ ©^Johan Svenson & Hans Christian Eilertsen, UiT

Several types of techniques are used for sample collections. Marine bacteria and microalgae are collected from both sediment and water samples using grabs and a CTD-rosette array offering enormous potential for biodiversity (Imhoff et al. 2011; Gulder and Moore 2009). The ability of marine bacteria to produce bioactive compounds has been recognised for more than 60 years (Rosenfeld and ZoBell 1947) and the first isolation of an antimicrobial compound (a pyrrole antibiotic, 2-(3,5-dibromophenyl)-3,4,5-tribromopyrrole) from a marine bacteria was reported in 1966 (Burkholder et al. 1966). As most marine organisms contain both exo- and endobiotic microorganisms it is generally difficult to establish the biosynthetic source of many marine natural products (König et al. 2006). There is growing evidence that the accompanying microflora is responsible for substantial amounts of the secondary metabolites discovered (Imhoff et al. 2011; Sudek et al. 2007). The Arctic marine bacteria have proven to be generally difficult to cultivate and only a minority can be grown on standard media in analogy to other marine bacteria (Jensen and Fenical 1994). Metagenomics can nevertheless be successfully applied to extract genetic information about promising cold-adapted proteins and secondary metabolites independent of cultivation (Simon and Daniel 2011; Godzik 2011; Kennedy et al. 2010; Sudek et al. 2007). Bottom-dwelling benthic organisms are collected through diving or several types of trawls. Each species is taxonomically identified and sufficient material is collected. Microbiological and genetic material from each species is also stored. Those microalgae and bacteria that can be cultivated are grown in the lab to provide enough biomass for isolation of secondary metabolites. This is the most sustainable way to get access to biological material but the generality is hampered by several factors discussed later (Fig. 4).

## Purification and screening

Once the biological sample has been gathered and sorted it needs to be analysed and that is the task of Marbio. Marbio is a high-throughput analytical laboratory with access to a range of automated and manual chromatographic and spectroscopic tools as well as a range of biochemical and cellular assays. Marine metabolites can be fractionated and identified and their discrete bioactivities evaluated. Before the bioactivities can be studied, the organism is lyophilised and extracted to yield an aqueous and organic (dichloromethane:methanol) extract. At least 200 g of biological material is used in an attempt to generate enough pure compounds to allow for spectroscopic structural elucidation. Both the organic and aqueous fractions are analysed. Each extract is separated on a flash chromatography system packed with HP20. 1 g of dried extract is dry loaded and eluted at a high flow (10–12 mL/min) using a gradient of water, methanol and acetone and fractionated into eight fractions.

The fractions are screened in several biochemical and cellular assays shown in Table 2. Only those displaying genuine dose–response behaviour upon dilution are further considered. The assays used are based on the needs of the commercial partners and on their clinical relevance. Major disease states such as cancer, infection, life-style diseases such as obesity, metabolic syndrome, and type-II diabetes are therefore targeted in addition to a more general search for antioxidants and immunomodulating compounds.

**Table 2:** MabCent screening targets

Activity	Target	Type
Toxicity	MRC-5 (normal lung fibroblast)	Cellular
Anticancer	HT-29 (colon adenocarcinoma), MCF-7 (breast cancer), A2058 (melanoma), IPC-81 (leukaemia)	Cellular
	NF-κB (A549)	Cellular
	Kinase inhibition	Biochemical
Antibacterial	*E. coli, S. aureus,* MRSA*, P. aeruginosa, E. faecalis*	Cellular
Biofilm	*S. epidermidis*	Cellular
Immunomodulation	TNF-α, IL-1β (THP-1), NF-κB (U937)	Cellular
Diabetes	PTB-1B	Biochemical
	PPARγ	Cellular
Antioxidants	FRAP	Biochemical
	ORAC	Biochemical
	Cellular Antioxidant Activity	Cellular

**Fig. 5:**
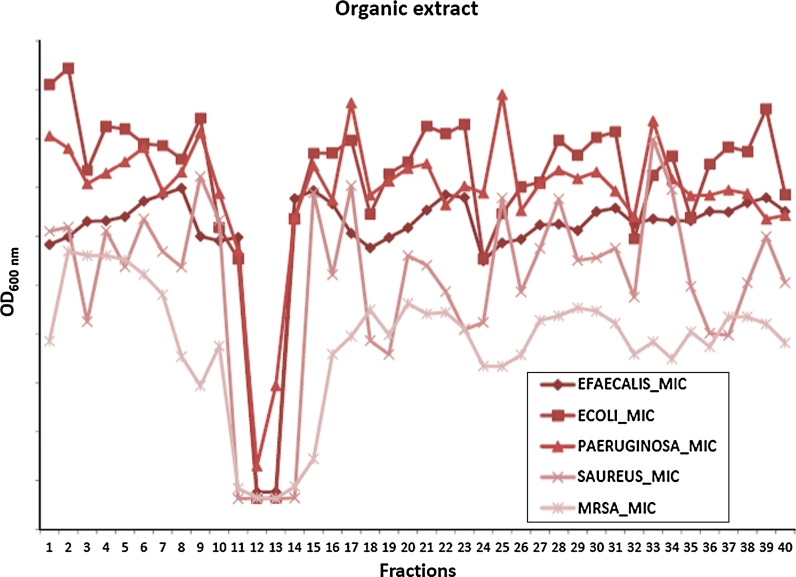
Antibacterial screening of 40 fractions from a RP-HPLC purification of a marine sample extract. High activity against both Gram positive and Gram negative bacteria is observed in fractions 10–14

Positive fractions (Fig. 5) are further purified by subsequent RP-HPLC separation steps to reduce the complexity of the active fraction and to pin the bioactivity to a single fraction. Once that has been achieved the task of identification ensues. While the strategy of MabCent is to look at marine organisms found in the Arctic, several of these have wider distributions. Consequently, most of the hits observed in the primary screening are caused by secondary metabolites that already have been discovered and described. Such a needle in the haystack challenge is only to be expected and identifying these known compounds as early as possible and removing them from biodiscovery pipeline is crucial. This dereplication process is on-going from the stage where the activity has been narrowed down to a single fraction.

High resolution mass spectrometry (HR-MS) analysis provides the first indication of novelty of the active compounds. The elemental composition, calculated from the accurate mass and the isotopic distribution is used to search databases such as the Dictionary of Marine Natural Products and AntiMarin before an isolation is attempted. By relying mainly on MS for the dereplication, significant time is saved as it does not require pure fractions or isolated compounds. Structural suggestions can to some extent be verified using MS^n^-fractionation experiments. Seemingly novel compounds are transferred to the structure determination platforms.

## Structure determination

The two platforms devoted to structure determination are in charge of the final structural trials. Macromolecules are handled by the Norwegian centre of structural biology (NorStruct) with particular experience and expertise in handling cold-adapted proteins. Cold adaption is crucial for survival at very low temperatures and is characterised by an increased molecular flexibility to compensate for the low surrounding temperatures. Such flexibility lead to an increased catalytic efficiency and a lower thermal stability (Smalas et al. 2000). Those traits permit the cold adapted enzymes to operate efficiently at low temperatures and it also allows for a simple way of controlling their activity, making them particularly interesting for molecular biology and biotechnological applications. X-ray crystallography is routinely used in conjunction with computational chemistry to study the proteins. NorStruct have characterised and studied several novel cold adapted enzymes, some of which have been transformed into commercial biotechnological and molecular biology products such as uracil-DNA glycosylase from Atlantic cod (*Gadus morhua*) (Leiros et al. 2003) and heat-labile shrimp alkaline phosphatase from *Pandalus borealis* (de Backer et al. 2002).

Smaller, more drug-like secondary metabolites that are suited for NMR analysis represent the task of SmallStruct. Access to cryoprobe fitted NMR-equipment enables analysis of the generally small sample amounts resulting from the purification and isolation processes. While the atomic connectivity of an unknown molecule is relatively easy to establish with NMR, the three-dimensional shape presents more of a problem. The low amount of pure compound often prevents the formation of crystals for X-ray analysis. Theoretical approaches in conjunction with chiroptical spectroscopic techniques, such as Raman optical activity, vibrational circular dichroism and electronic circular dichroism are therefore under development together with the Norwegian centre of theoretical and computational chemistry. This allows in-solution determination of absolute configuration in cases where X-ray analysis is not a feasible option. This approach was recently employed to the synoxazolidinones to reveal their absolute configuration (Hopmann et al. 2012). SmallStruct is further involved in the synthetic aspects of MabCent and are performing analogue synthesis and development of the most interesting leads. The group has particular experience from the field of naturally occurring bioactive peptides and peptidomimetics (Svenson et al. 2008; Karstad et al. 2010; Flaten et al. 2011).

## Hits so far

**Fig. 6:**
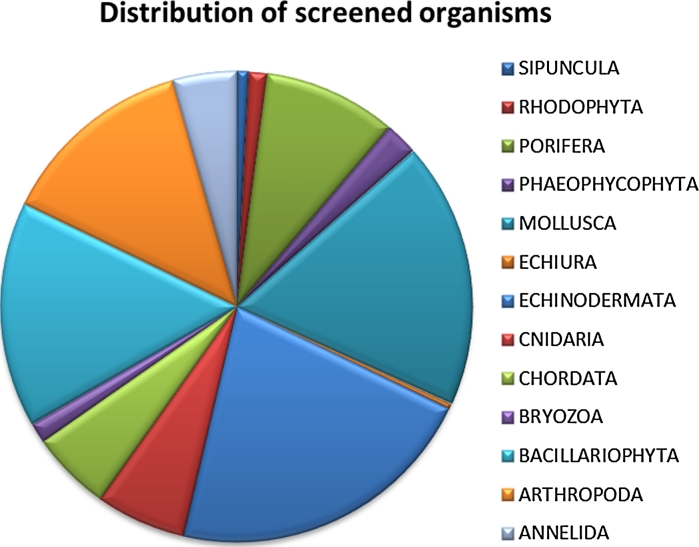
Pie chart diagram over the distribution of the organisms screened by Marbio. The majority of the organisms are of benthic origin with microalgae, diatoms (*Bacillariophyta*) being the only class of microorganisms screened so far

So far more than 25,000 fractions from 140 different marine organisms (mostly benthic) have been analysed and numerous bioactive marine compounds have been isolated and characterised. A schematic representation of the distribution and origin of the analysed organisms is shown in Fig. 6.

**Fig. 7:**
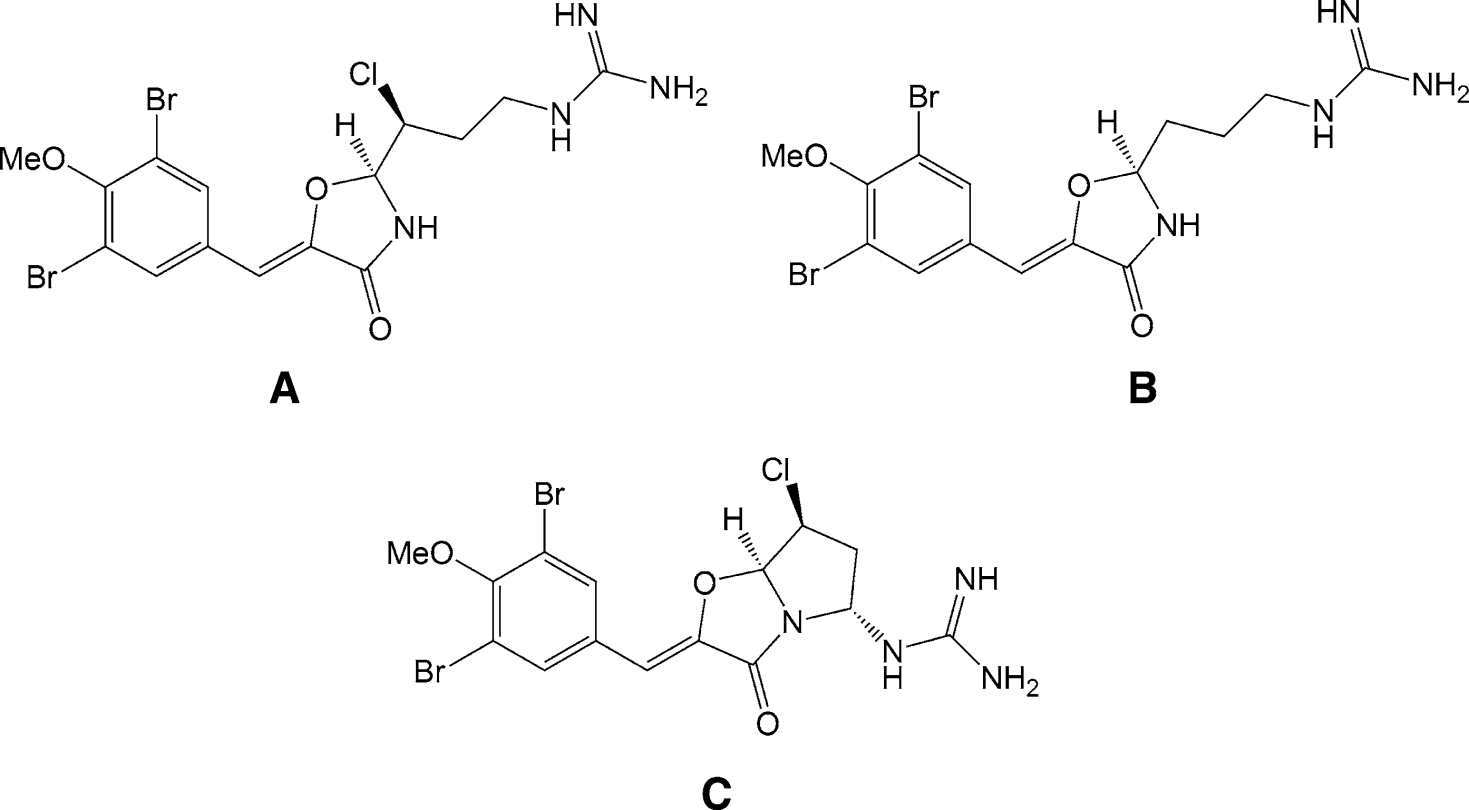
Synoxazolidinones (**A-C**)

The vast majority of the compounds isolated are however already known ones and represent dereplication rather than pure discoveries as the majority of the species studied and their accompanying microflora have a large geographical distribution. Of the novel compounds discovered, the antibacterial, antifungal and cytotoxic synoxazolidinone (Tadesse et al. 2010; Tadesse et al. 2011a) family stands out (Fig. 7) and they are being further developed by the commercial partner Lytix Biopharma AS.

**Fig. 8:**
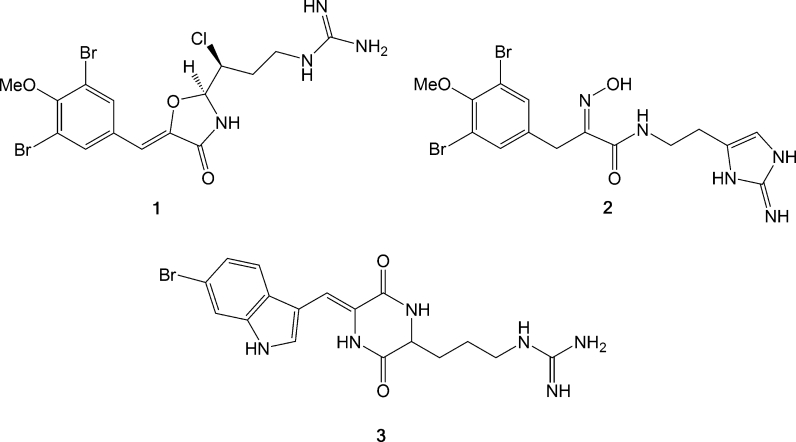
Many marine secondary metabolites are derived from heavily post-translationally modified dipeptides and several such compounds have been identified as a result of the bio-assay guided fractionation at Marbio. Synoxazolidinone A (**1**), ianthelline (**2**) and barettin (**3**) are three such compounds with several chemical and structural similarities

This is a family of closely structurally related compounds that was initially isolated in surprisingly high concentrations from the sub-Arctic ascidian *Synocium pulmonaria.* The synoxazolidinones contain a highly unusual heterocyclic oxazolidinone core linking two amino acid (Arg and Tyr) derivatives together. They represent only the second report of a naturally occurring 4-oxazolidinone motif with the highly antibacterial lipoxazolidinone family being the first one (Macherla et al. 2007) reported. The motif is also shared with the novel synthetic 50S ribosomal subunit binder antibiotic linezolid (Moellering 2003). It has not been established whether the biosynthetic origin of the synoxazolidinones is the ascidian itself or symbiotic microorganisms. Such brominated dipeptidic derivatives are regularly produced by marine organisms and the synoxazolidinones share structural traits with for example barettin (Lidgren and Bohlin 1986) from the cold-water sponge *Geodia baretti* and ianthelline (Litaudon and Guyot 1986) found in the tropical *Ianthella ardis* as shown in Fig. 8.

**Fig. 9:**
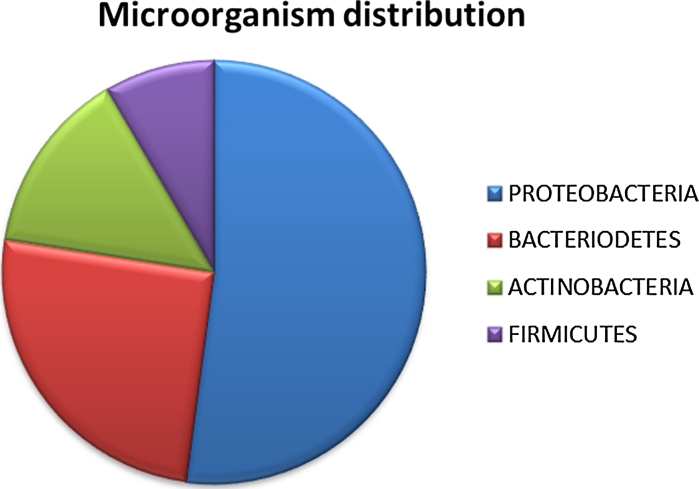
Pie chart diagram of the phyla distribution of the microorganisms collected by Marbank

In addition, the several recently described new members of the antibacterial eusynstyelamide family also share this structural composition of halogenated hydrophobic bulk and one or two cationic groups (Tadesse et al. 2011b). A clear correlation between bioactivities and the nature of the cationic side chains were seen for these novel compounds (Tadesse et al. 2011b). Currently, numerous novel bioactive compounds are undergoing structural elucidation and the annual number is expected to increase the coming years due to the improved efficiency of Marbio and the introduction of both bacteria and on-site grown microalgae into the screening process. Nearly 1500 isolates of microorganisms have been collected and 16S sequenced and they have yet to enter the pipeline which has so far been nearly exclusive for benthic organisms with the exception of the microalgae. The bulk of the microorganisms belong to the *Proteobacteria* and *Bacteriodetes* phyla as shown in Fig. 9. The collected microorganisms have further been taxonomically divided into 500 species and 70 different families.

## Discussion

MabCent has been up and running since 2007 and will continue at least until the end of 2014 in its current form. Despite the fact the project is now on its fifth year there are still activities that can be improved and the learning curve for most involved staff has been steep and several lessons have been learnt the hard way. Initially, the progress was hindered by idiosyncratic false positives and active fractions without any apparent content. A change from an initial 40 fraction HPLC fractionation to an early flash chromatographic separation and a proper dose–response analysis of the active hits reduced those numbers to satisfactory levels.

One of the most challenging issues is, despite the geographical placement of MabCent, access to sufficient amount of biological material and subsequently pure compounds. This is a universal challenge for any marine bioprospecting endeavour, perhaps best illustrated by the 18 grams of bryostatin-1 needed for preclinical and clinical studies in 1991 which was isolated from approximately 12.6 metric tonnes of wet organism over a period of 10 months (Schaufelberger et al. 1991). Multi tonne amounts were also needed for the isolation of sufficient material of halichondrin (Hirata and Uemura 1986) and the approved antitumoral ecteinascidin 743 (Trabectidin/Yondelis^®^) which had its final structural elucidation delayed by two decades due to insufficient amounts (Molinski et al. 2009). A low amount of starting material is thus an undisputed way to miss out on the most active compounds as they are by definition not needed in the same amounts as other metabolites. The antimitotic macrolide halistatin-1 for example is only present at 8.8 parts per billion in the organism (Pettit et al. 1993) illustrating that attention to the “small peaks” can be crucial. This has been obvious several times in the MabCent screening where seemingly empty HPLC fractions generate positive results in the screening assays. MS-analysis sometimes later reveals the presence of potentially novel compounds in the fractions but in no-way near high enough amounts for characterisation. The 200 grams of organism generally used by MabCent for the initial extraction may appear insufficient, but already at this stage, that minimum amount prevents analysis of numerous collected organisms. Those highly active compounds can also cause initially perplexing results upon purification of active fractions of seemingly high molecular content. For example: A 1 nM agonist present at a 1 % concentration in a mixture with 99 % of another inactive compound will provide the mixture with a promising IC_50_ of 0.1 μM. The agonist itself will nevertheless most likely be regarded as noise or as “an acceptable amount” of contamination in the initial NMR analysis of the major inactive compound in that mixture thought to generate the bioactivity. Further purification removing the actual agonist will yield a pure major product with no bioactivity whatsoever in the final dose–response analysis. Such scenarios are particularly prone to appear when working with automated high-throughput techniques.

Only the most common benthic organisms and macroalgae are readily available on demand and these are also frequently the species already most thoroughly studied by others. Gathering Arctic organisms far out at sea in a dark, cold and hostile environment is not for the faint hearted and is not to be compared with a reef walk collection at low tide on a tropical coral reef. For this reason it is difficult to resample an already visited location to stock up on more of the organism if the initial results are encouraging and more material required. Seasonal variation also influences the production of secondary metabolites and it has been shown that the same species collected at the identical location a year later does not necessarily contain the same compounds (Molinski et al. 2009; Tadesse et al. 2010). So in short, one must make sure to collect as much as possible while sampling and only allow the freezer capacity on the vessel to be the limiting factor. Such an approach raises questions about sustainability (Bohlin et al. 2010) and it is clear that Nature will, only as an exception, be able to provide enough material once a commercially viable compound has been identified. If the producing organism cannot be aquafarmed, or if the compound itself cannot be synthesised, or produced by microorganisms on a large scale then one is faced with plummeting odds for success after the initial discovery.

**Fig. 10:**
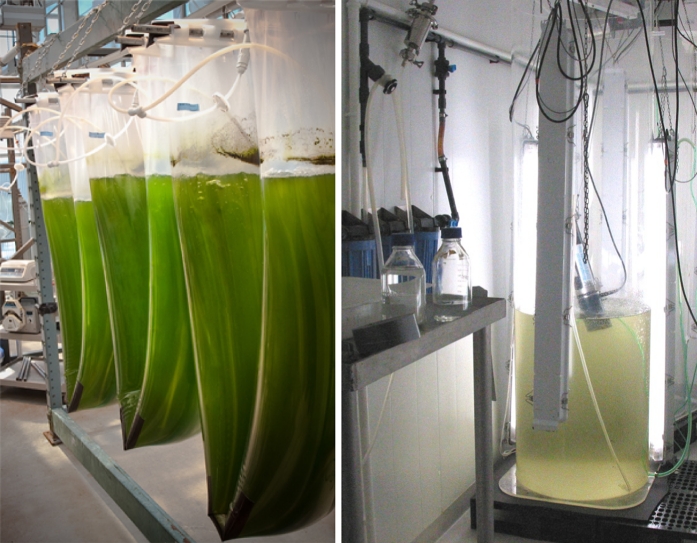
Focus on organisms that can be grown in a controlled fashion is of high priority for both sustainability and practical reasons. Diatoms can for example be readily produced in both bags (*left*) or large scale cylinder incubators (*right*). Arctic microalgae are flexible and high producing at low light, temperature and nutrition (N&P).^ ©^Hans Christian Eilertsen & Richard Ingebrigtsen UiT

Aware of these hurdles, MabCent has dedicated significant resources into the development of methods to grow microalgae on a large scale. The experiments have so far been successful and several species of diatoms can now be readily grown in 600 L incubators in high concentrations as shown in Fig. 10.

Screening of these organisms has just begun and it is realistic to expect that this strategy will generate rapid access to a renewable source of biological material. Extra attention to growth conditions must nevertheless be paid as most of the secondary metabolites serve as defence mediators and their production may be lowered or stopped when grown in a monoculture without competing species or predators providing a selection pressure. This has previously been shown for the alga *Phaeocystis pouchetii*, that only upon grazing releases toxic aldehydes to fend off the intruders (Hansen et al. 2004). The “suicide” defence mechanism, triggered by being consumed, of the diatom *Thalassiosira rotula* is another example (Pohnert 2002) illustrating that carefully combined cultures may be needed to generate biomass that is also containing the desired compounds. The off-shore harvest and large scale in-shore aquaculture of the sponge *Lissodendoryx* n. sp. to eventually produce 310 mg of halichondrin B illustrate that with sufficient financial support it may also be possible grow relevant marine macroorganisms (Munro et al. 1999; Molinski et al. 2009).

Most of the MabCent pipeline is designed for high-throughput and automation (Luesch 2006). This generally works satisfactory for the assays but is less effective for the fractionation process. The challenges associated with high-throughput and natural product extracts are established and several technological improvements are under development (Koehn and Carter 2005). The purification has benefited from applying extra manual manpower and attention to this stage of the process. Ideally each fraction should be followed by a single scientist but that is not a realistic approach given the number of species collected and the timeframes of the project. MabCent is funded for 8 years and its ambition is to screen as many Arctic marine organisms as possible within that period. Such ambitions can only be achieved using a certain degree of high-throughput and automation and it may also potentially mean that some of the low concentration/high affinity compounds could pass through the system without the attention they need to be discovered. All the data from the screens are however stored and extracts with high potencies but no or low apparent content can be backtracked and re-analysed later once the “lowest hanging fruits” have been studied.

## Outlook

The MabCent project has now passed halfway and the results of the final 3 years will most likely influence the future direction of research within this field in Norway. The MabCent operation has survived the first difficult years and is now running smoothly and productively and the expectations remain high. With the microorganisms now entering the screening stage there is a general belief that the number of novel compounds will increase.

Several groups at other Norwegian universities have independently started to look into marine bioprospecting and marine chemistry but they are still in the initial start-up phases. Norway is a small country and a national strategy has been developed to generate synergies and make the most of the national resources and competences to allow for future focus on this field.
